# Gastro-Esophageal Junction Precancerosis: Histological Diagnostic Approach and Pathogenetic Insights

**DOI:** 10.3390/cancers15245725

**Published:** 2023-12-06

**Authors:** Cinzia Giacometti, Anna Gusella, Mauro Cassaro

**Affiliations:** Pathology Unit, Department of Diagnostic Services, ULSS 6 Euganea, 35131 Padova, Italy; anna.gusella@aulss6.veneto.it (A.G.); mauro.cassaro@aulss6.veneto.it (M.C.)

**Keywords:** intestinal metaplasia, Barrett’s dysplasia, esophageal dysplasia

## Abstract

**Simple Summary:**

A diagnosis of Barrett’s esophagus (BE) requires the macroscopic visualization of gastric-appearing mucosa in the esophagus and the identification of intestinal metaplasia on histologic examination. Histologic diagnosis of BE dysplasia can be challenging due to sampling error, pathologists’ experience, interobserver variation, and difficulty in histologic interpretation: all these problems complicate patient management. In intestinal metaplasia, which occurs because of chronic gastresophageal reflux disease (GERD), the squamous epithelium converts to columnar epithelium, which is initially of the cardia type and devoid of goblet cells; it later develops goblet cell metaplasia and eventually dysplasia, which develops and progresses to adenocarcinoma because of the accumulation of multiple genetic and epigenetic alterations. Therefore, this review aims to provide an up-to-date and clear diagnostic approach to Barrett’s esophagus and an overview of dysplasia’s development’s pathogenetic and molecular mechanisms.

**Abstract:**

Barrett’s esophagus (BE) was initially defined in the 1950s as the visualization of gastric-like mucosa in the esophagus. Over time, the definition has evolved to include the identification of goblet cells, which confirm the presence of intestinal metaplasia within the esophagus. Chronic gastro-esophageal reflux disease (GERD) is a significant risk factor for adenocarcinoma of the esophagus, as intestinal metaplasia can develop due to GERD. The development of adenocarcinomas related to BE progresses in sequence from inflammation to metaplasia, dysplasia, and ultimately carcinoma. In the presence of GERD, the squamous epithelium changes to columnar epithelium, which initially lacks goblet cells, but later develops goblet cell metaplasia and eventually dysplasia. The accumulation of multiple genetic and epigenetic alterations leads to the development and progression of dysplasia. The diagnosis of BE requires the identification of intestinal metaplasia on histologic examination, which has thus become an essential tool both in the diagnosis and in the assessment of dysplasia’s presence and degree. The histologic diagnosis of BE dysplasia can be challenging due to sampling error, pathologists’ experience, interobserver variation, and difficulty in histologic interpretation: all these problems complicate patient management. The development and progression of Barrett’s esophagus (BE) depend on various molecular events that involve changes in cell-cycle regulatory genes, apoptosis, cell signaling, and adhesion pathways. In advanced stages, there are widespread genomic abnormalities with losses and gains in chromosome function, and DNA instability. This review aims to provide an updated and comprehensible diagnostic approach to BE based on the most recent guidelines available in the literature, and an overview of the pathogenetic and molecular mechanisms of its development.

## 1. Introduction

When assessing the efficacy of interventions for any medical condition, it is crucial to take into consideration the natural progression of the condition. In the case of Barrett’s esophagus (BE), the emergence of esophageal adenocarcinoma (EAC) is the most significant and relevant outcome. In Western countries, BE—the only recognized precursor of EAC—affects 2–7% of adults [1,2], and over the last decade, there have been significant advances in the comprehension of biology and pathology of the esophagus and GEJ in response to injury sustained as a result of chronic gastresophageal reflux disease (GERD) [3], even if some studies revealed that BE prevalence is also substantial in patients without GERD [4] It is believed that BE progresses to EAC in stages, with dysplasia (low-grade—LGD and high-grade—HGD) occurring before the development of EAC. It is crucial to monitor patients with recognized and established BE to prevent the development of EAC. Some data suggest that surveillance can help improve patients’ outcomes [5]. There is significant variability in the reported rate of progression of LGD, which is mainly attributable to the significant differences in the way LGD is classified by pathologists [6], so the risk of progression for LGD depends on the accuracy of its diagnosis. The diagnosis may vary depending on the experience and expertise of the local practitioners: the diagnosis of LGD in community centers can be unreliable. Therefore, when the diagnosis of LGD is made, it is recommended that biopsies should be repeated and examined by at least two expert gastrointestinal pathologists. If LGD is conclusively diagnosed, the annual risk of progression to cancer may be around 1–3% [7,8,9]. The progression rate from LGD to HGD/EAC is higher and is estimated in about 5–10% [10]. Even if the diagnosis of HGD is more straightforward, HGD can be easily over-diagnosed [11].

Endoscopic surveillance and treatment for Barrett’s esophagus (BE), LGD, HGD, and EAC rely heavily on the accuracy of histological diagnosis. To improve this accuracy of the histological diagnosis and shed light on the molecular mechanisms of BE, we conducted a thorough literature review.

## 2. On the Existence of Cardiac Mucosa

**Figure 1:**
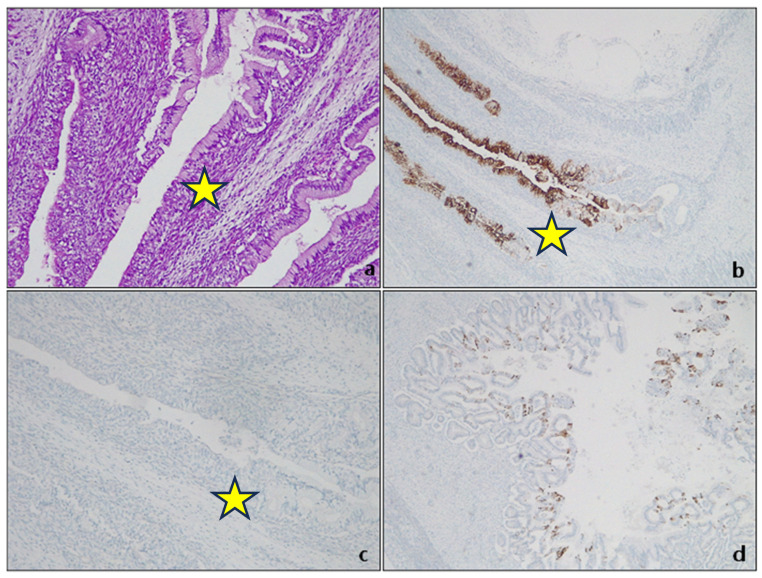
Original autopsy fetal case, 15 weeks of gestation. GEJ (yellow star). (**a**) A transition zone of mucous glands between the proximal fundic gastric gland and the multilayered epithelium is abrupt. H&E. Original magnification 200×; (**b**) CK7 expression. Original magnification 25×; (**c**) absence of CK20 expression in cardial mucosa. Original magnification 100×; (**d**) CK20 expression in pyloric mucosa. Original magnification 100×.

The esophageal mucosa comprises a nonkeratinizing superficial stratified squamous epithelium, a lamina propria containing scattered mucous glands, and mixed mucous/serous glands resembling salivary glands in the submucosa. The gastric mucosa comprises mucinous columnar epithelium, with pure oxyntic or pure mucous glands in the deeper portion of the body’s mucosa and antrum, respectively. The passage between the antrum and the body is usually composed of a mixture of oxyntic and mucous glands [12]. The gastric cardia is assumed to be the most proximal part of the stomach, defined as that portion of the mucosa (1–4 mm) between the GEJ (which is the proximal limit of the gastric folds) and the gastric body, entirely composed of oxyntic glands [13,14]. Many attempts have been made in recent years to clearly define (anatomically, endoscopically, and histologically) this peculiar region to better understand the pathophysiology of the observed metaplastic changes, in particular Barrett’s esophagus and their association with dysplasia (of low and high grade) and the possible evolution into adenocarcinoma. The squamocolumnar junction (SCJ) is a peculiar transition zone encountered in different human body districts: the uterine cervix, anorectal passage, and gastresophageal junction (GEJ). In all cases, this is the site of epithelial modifications due to infectious factors (Human papillomavirus—HPV, Helicobacter pylori—HP) and inflammatory factors (such as bile or acid reflux, especially in the context of GERD) [15,16,17]. Unlike other sites, the SCJ is not as easily described and identified in the upper gastrointestinal tract. Between 1946 and 1956, Allison and Barrett attempted to define the final part of the esophageal mucosa [18,19,20,21], leading for the first time to the concept that the distal portion of the esophagus, lined by an epithelium similar to the gastric antrum, could be the consequence of exposure of the esophageal squamous epithelium to gastric acid reflux. As Allison wrote: “Consideration of the healing process raises a further problem: if gastric reflux occurs, which causes ulceration of the squamous epithelium without producing strictures, it is more likely that the healing of the ulcer in an acidic environment is gastric rather than esophageal epithelium? If so, it could be that some examples of gastric mucosa in the esophagus were acquired rather than congenital” [19]. The transition point separating the whitish esophageal squamous mucosa from the reddish gastric columnar mucosa (SCJ), known endoscopically as the Z-line, is a crucial landmark for assessing gastric mucosal fold length and describing signs of reflux acid [22]. The definition, extent, and histological features of the GEJ region are still controversial. Some autopsy studies on the gastresophageal junction (GEJ) in embryos and fetuses suggest that the cardiac mucosa is a normal structure present at birth, according to De Hertogh et al. [14]. However, there is debate as to whether a mucosa composed of pure mucous cells exists during normal fetal development in the transition from stomach to esophagus, as argued by Park et al. [15]. In fetal tissue at 15 weeks of gestation, the glandular epithelium expresses CK5/6, CK7, CK8/18 (differently from the oxyntic mucosa of the gastric fundus) and CK19, the latter being similar to the gastric oxyntic mucosa, but not CK20 (Figure 1). 

**Figure 2:**
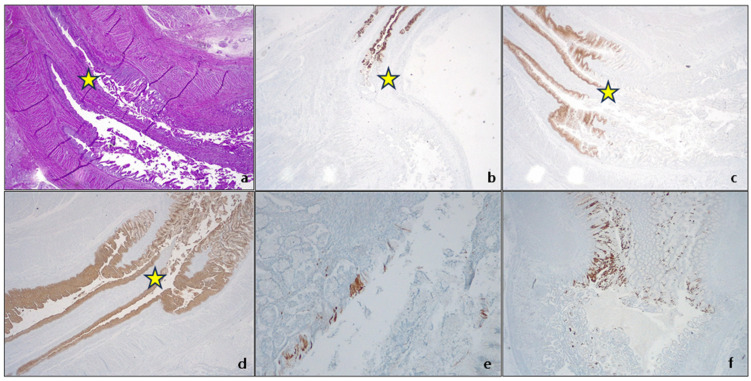
Original autopsy fetal case, 21 weeks of gestation. GEJ (yellow star). (**a**) The transition zone of mucous glands between the proximal fundic gastric gland and the “squamous” stratified epithelium is abrupt. H&E. Original magnification 200×; (**b**) CK7 expression. Original magnification 25×; (**c**) CK5/6 expression. Original magnification 25×; (**d**) CK 19 expression. Original magnification 25×; (**e**,**f**) CK20 expression in cardial (**e**) and pyloric (**f**) mucosa.

CK19 is the most represented CK in the foregut embryonic epithelium, together with CK7, which is present especially in the columnar cell layer, while CK 20 is absent in the columnar epithelium of the esophagus and GEJ and is typically and focally present in the gastric antrum [23]. Later in the fetal life (after 17 week of gestation), CK20 is also expressed in the surface of esophageal columnar epithelium [23]. At 21 weeks of gestation, the CKs pattern of expression has changed, and the glandular epithelium acquires a phenotype similar to the pyloric glands (Figure 2). 

The pattern of keratin expression in the transitional mucosa of the fetal gastresophageal junction shows interesting similarities to the adult cardiac mucosa. CK expression patterns suggest a cardial-type mucosa is present during fetal life and is established before birth. This could indicate that the adult cardial mucosa has a congenital origin, at least partly [16]. The anatomical gastresophageal junction (GEJ) aligns with the Z line histologically in individuals with no medical conditions. Many adults with GERD, central obesity, or hiatus hernia have a proximally displaced or irregular Z line, causing the histological SCJ to lie above the anatomical GEJ. This segment of the mucosa above the anatomical GEJ may be composed of pure or mixed mucous/oxyntic glands. Biopsies taken from this site could be misinterpreted as the “true” gastric cardia while representing the metaplastic columnar epithelium of the distal esophagus [24,25,26,27].

## 3. Barrett’s Esophagus

### 3.1. Guidelines and Definitions: A Long Journey to Standardization

Difficulties in unambiguously establishing the exact location and origin of SCJ, EGJ, and cardia are responsible for differences in definitions and diagnosis of BE worldwide. The endoscopic diagnosis of BE is made by recognizing a velvet-like, salmon-colored mucosa in the distal esophagus, which is in continuity with the gastric folds. Thus, the endoscopic defining of the landmark of GEJ constitutes a significant difference between the guidelines regarding the endoscopic diagnosis and pathological findings of BE and follow-up methods for the early detection of Barrett’s esophageal adenocarcinoma. Many have been published (or revised) around the world in the last few years: in Europe by the European Society of Gastrointestinal Endoscopy (ESGE) and the British Society of Gastroenterology (BSG); in the United States by the American Society for Gastrointestinal Endoscopy (ASGE), the American Gastroenterological Association (AGA), and the American College of Gastroenterology (ACG); in Japan by the Japanese Esophageal Society (JES); in the Asian-pacific area by the Asian Pacific Association of Gastroenterology (APAGE).

As the endoscopic diagnostic methods have not been standardized, each guideline differs from the other (Table 1) regarding the length of columnar epithelium, endoscopic landmark, and the requirement of intestinal metaplasia to define BE [28,29,30,31,32,33].

During the recent Kyoto international consensus meeting, some significant issues were addressed to standardize the diagnosis of BO worldwide. As an endoscopic landmark, the consensus meeting adopted DEPV as more appropriate for defining GEJ since it has a more valid anatomical basis than the landmark [34]. 

### 3.2. Intestinal Metaplasia, or Not: Still a Diagnostic Requirement?

**Figure 3:**
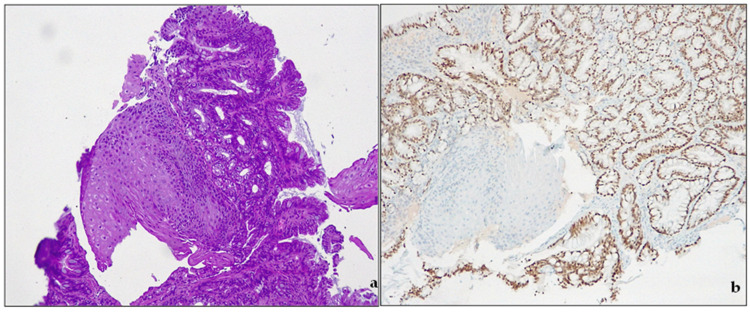
Barrett’s esophagus. (**a**) intestinal metaplasia. H&E, original magnification 100×; (**b**) CDX2 expression, original magnification 100×.

Metaplasia is classically defined as “the conversion, during postnatal life, of one differentiated cell type to that of another” [35]. The concept of metaplasia deals with three main notions: (a) normal tissue is located in the wrong place; (b) that tissue appeared in the wrong place during postnatal life; and (c) the presence of that tissue in that wrong place is due to its actual development there, rather than its migration from an adjacent area [36]. By these definitions, true metaplasia is only that which derives from cellular transformations due to changes in developmental commitment. In human pathology, metaplasia is usually associated with tissue chronic damage and arises most often in regenerating tissues. Chronic damages, metaplasia and cancer development are a well-known pathway in clinical settings: BE is one of them [37]. In the debate around the definitions of BE, intestinal metaplasia (IM) plays a pivotal role. IM (Figure 3) indicates the presence of a specialized (intestinal) metaplastic tissue in the distal esophagus. IM is a known risk factor for developing gastric or esophageal cancer. 

However, some scientific societies (BSG, APAGE, JES) do not consider the presence of histologically detected IM to diagnose BE (Table 1). This position relies on the demonstration that cardiac mucosa, considered the normal lining of the gastric cardia, also found in embryos and fetuses [38,39,40], can also have intestinal-type histochemical features and abnormalities in DNA content, it appears to be a GERD-induced change, and it is the substrate for the development of GEJ adenocarcinoma [41].

The Kyoto International Consensus Meeting discarded IM for the assessment of BE histological diagnosis [34]. 

### 3.3. Molecular Pathway: From Normal Tissue to Metaplasia: Transdifferentiation, Transcommitment and Cell of Origin

A possible explanation for BE metaplasia is transdifferentiation, where fully differentiated esophageal squamous cells transform into fully differentiated columnar cells (Campo, 32). In Barrett’s esophagus, differentiated cells can regress to become immature progenitor cells, leading to increased expression of genes such as CDX1 and c-Myc, compared to the normal epithelium of the esophagus (Campo, 33). CDX2, a transcription factor belonging to the caudal-related homeobox gene family, is thought to be an early marker of intestinal differentiation and may play a role in the development of intestinal metaplasia (Campo, 34). Transdifferentiation in the esophagus may occur in two stages: first, completely differentiated, mature, squamous cells acquire progenitor cell-like plasticity; secondly, they can re-enter the cell cycle to repair injured tissues by transforming into a metaplastic tissue that might be more resistant to GERD. These cycles may repeat over time. Murine models with induced gastric HP infection showed a complex pattern of peptide expression, such as trefoil factor 2 (TFF2). During the answer to the injury, the cells silence mTORC1 signaling, enabling autophagy to recycle cellular material to synthesize new structures. After that, they express genes associated with metaplasia, such as SOX9 and TFF2, and then reactivate mTORC1 signaling to re-enter the cell cycle. Trefoil proteins may be important in protecting and repairing gastrointestinal tract [41,42]. 

In the 1990s, some investigators discovered the multilayered epithelium (MLE), a peculiar histologic entity observed at the SCJ. It comprised four to eight layers of stratified squamous cells at the bottom of of mucin-containing epithelial cells with microvilli. These cells are different from Barrett’s intestinalized and normal esophageal squamous cells [37,38]. This MLE is not visible in intestinal metaplasia that is related to chronic gastritis. Also, its immunohistochemical CK7, CK14, CK19, and CK20 expression pattern is similar to the epithelium of the esophageal gland duct. Some data suggest that the preservation of CK7 and the loss of CK20 expression occurs in the progression of metaplasia to dysplasia and carcinoma, indicating a de-differentiation towards a progenitor cell phenotype Campo [39,40]. The process by which immature progenitor cells that can proliferate and differentiate into different cell types are reprogrammed to alter their regular differentiation pattern is transcommitment [31]. There are four categories of candidates that could be progenitor cells that give rise to Barrett’s metaplasia: (1) progenitor cells native to the esophagus (including basal cells of the squamous epithelium or cells of esophageal submucosal glands and their ducts); (2) progenitor cells native to the gastric cardia that might migrate into the esophagus in response to reflux damages; (3) specialized populations of cells at the esophagogastric junction (EGJ). (4) bone marrow progenitor cells [41].

## 4. Dysplasia in Barrett’s Esophagus

### 4.1. Definition

Barrett dysplasia is defined by a morphologically unequivocal neoplastic epithelium without invasion, occurring in an area of metaplastic columnar epithelium in the esophagus [43]. Endoscopically, dysplasia is usually associated with an irregular pit pattern when analyzed with NBI magnification [44,45].

### 4.2. Type of Dysplasia 

#### 4.2.1. Intestinal-Type Dysplasia

Intestinal dysplasia is a common form of dysplasia in BE, and it resembles a typical colonic adenoma. It is made up of columnar cells that have intestinal differentiation and goblet cells. In contrast, foveolar or non-intestinal gastric dysplasia demonstrates glands closely packed with a single layer of columnar cells and few or no interspersed goblet cells. The basal nuclei are round or oval with little stratification or pleomorphism, and the nuclei are vesicular with prominent nucleoli [3,46].

**Figure 4:**
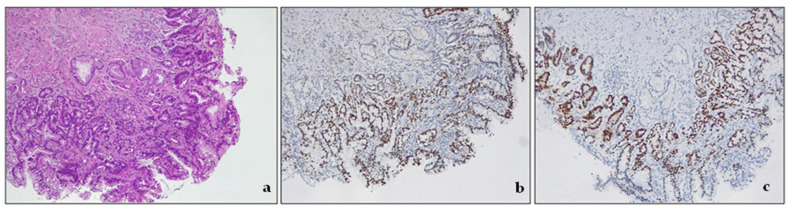
Intestinal type dysplasia, low grade. (**a**) The glands are packed, but lamina propria is still detectable between the glands. There are some scattered residual goblet cells. H&E. Original magnification 200×; (**b**) CDX2 expression in metaplastic glandular epithelium, devoid of goblet cells. Original magnification 200×; (**c**) p53 expression in metaplastic glandular epithelium, devoid of goblet cells. Original magnification 200×.

Low-grade dysplasia (LGD) is a pathological condition characterized by cells with nuclear enlargement, elongation, hyperchromasia, and stratification but with retained nuclear polarity (Figure 4). The dysplastic crypts typically show minimal architectural changes, and there is still evident lamina propria between them. The nuclei are slightly enlarged, and the number of mitoses generally increases, but they still appear normal. The cytoplasm is usually eosinophilic and depleted of mucin. The number of goblet cells may vary from very few to numerous [3,43]. 

High-grade dysplasia (HGD) is characterized by significant cytological atypia and widespread architectural changes. The cells have enlarged nuclei, often up to three to four times the size of lymphocytes, with full-thickness nuclear stratification in both the base and surface epithelium. There is also nuclear pleomorphism, irregular nuclear contours, and a substantial loss of polarity. Mitotic activity is increased, and atypical mitoses are frequently observed. Additionally, intraluminal necrosis may be present. The crypts in high-grade dysplasia may vary in size and shape, appear crowded, or contain marked budding, angulation, back-to-back growth, and cribriforming. The diagnosis of HGD can be made based on either high-grade cytological or architectural aberrations. However, most cases show a combination of both cytological and architectural abnormalities [41].

#### 4.2.2. Non-Intestinal Dysplasia

Foveolar dysplasia, which is non-intestinal, does not exhibit any cytologic characteristics of intestinal differentiation. It demonstrates mucinous (also known as “foveolar”) cytoplasmic changes, frequently with a complete absence of goblet cells. Morphologically, it resembles the gastric foveolar epithelium [44]. Foveolar dysplasia appears as an epithelium composed of cells with abundant mucinous cytoplasm and only a few goblet cells. The cellular composition consistently displays a homogeneous, singular layer. Their size is relatively small or moderately enlarged, featuring round to oval-shaped nuclei positioned at the base with no discernible fluctuations in size or shape. The surface epithelium is invariably implicated, while the bases of the crypts may be spared. Identifying LGD foveolar type can be challenging as it closely resembles non-neoplastic, gastric cardia mucosa, particularly in an inflammatory backdrop [47]. 

In one study by Khoer et al., foveolar dysplasia was found to be highly associated with a complete gastric immunophenotype (MUC5A+, MUC2−), while “classic” adenomatous/intestinal dysplasia was associated with a complete intestinal immunophenotype [48]. 

HGD is characterized by large, round to oval nuclei, open chromatin pattern and prominent nucleoli, and increased mitoses. There is no significant loss of polarity, stratification, and pleomorphism. Even in HGD, the cells tend to retain a regular appearance. The most striking feature is architectural: the crypts are more compact, elongated and show extensive branching and complexity without intervening lamina propria [3]. 

### 4.3. Diagnostic Categories

In accordance with growing evidence that BE was a complex entity, Reid et al., in 1988, via a preliminary consensus conference, defined a five-tiered histologic classification of BE’s dysplasia: 1. negative for dysplasia; 2. indefinite for dysplasia; 3. low-grade dysplasia; 4. high-grade dysplasia; 5. Intramucosal carcinoma [49]. Ten years later, in order to “develop a common worldwide terminology for gastrointestinal epithelial neoplasia”, in 1998, a consensus meeting was held in Vienna [50]. The Vienna classification is still in use [43] and pathologists should report dysplasia according to the four diagnostic categories proposed: 1. negative for dysplasia; 2. indefinite for dysplasia; 3. low-grade dysplasia; 4. high-grade dysplasia (Table 2). The rationale for this tiered approach is to stratify patients into categories of increasing risk for the development of esophageal adenocarcinoma (EAC).

1. Negative for dysplasia. This diagnosis is made when the biopsy represents either columnar epithelium with no cell atypia or reactive (hyperplastic/regenerative) changes.
2. Indefinite for dysplasia. This category reflects the uncertainty of the diagnosis. As the real nature of the lesion cannot be assessed, follow-up should be suggested in the report [50]. In some settings, biopsy interpretation can be highly challenging for pathologists. Active inflammation, ulceration, or post-ulcer healing may determine profound changes in tissue. This descriptive, provisional category should apply only to cases where the pathologist cannot clearly decide whether the lesion is negative for dysplasia (hyperplastic/regenerative) or genuinely dysplastic. The grade of uncertainty may be due to inadequate biopsy sampling or cytological atypia and structural alterations with equivocal interpretation. This diagnosis must be followed by short-term resampling and second opinion and not be used as a “waste-basket” category.
3. Low-grade dysplasia—LGD. The cells in LGD display nuclear enlargement, elongation, hyperchromasia, and stratification, but their nuclear polarity is retained (Figure 4). Although the dysplastic crypts show minimal architectural changes, the lamina propria between them is still visible. The nuclei are slightly enlarged, and the number of goblet cells present may range from a few scattered ones to numerous.
4. High-grade dysplasia—HGD. HGD is characterized by striking cytological atypia and wider architectural changes. The cells have markedly enlarged nuclei, nuclear pleomorphism, irregular nuclear contours, and loss of polarity. Mitoses are increased in number and are often atypical. The crypts may appear crowded, and/or may contain marked budding or angulation, back-to-back growth, and cribriforming.

### 4.4. Ancillary Techniques

Some ancillary diagnostic markers, mainly immunohistochemical, have been investigated to facilitate BE diagnosis: proliferation markers such as Ki67, genetic mutations (p53, p16, Kras, APC, B catenin), some growth factors, cyclooxygenase 2 (COX-2), and alpha-methylacyl-CoA racemase (AMACR). p53 is a transcription factor expressed by the tumor suppressor gene TP53 (chromosome 17p). The TP53 gene encodes p53, which prevents mutations. Normal cells (wild type) have low levels of this protein in their nuclei. The gene and protein are upregulated in the presence of DNA damage or stress. In dysplastic cells, mutations of TP53 can be detected by IHC, which may show a different pattern of expression: (a) complete loss, absent (null) pattern, completely negative staining; (b) cytoplasmic patter; (c) mutation pattern, with increased expression and positive staining. Recently, Redston et al. proposed a scoring method for p53 expression in BE [51,52,53]. Even if promising, p53 provides variable results (both false positive and false negative): its use to diagnose dysplasia in clinical practice is still controversial. More recently, biomarkers different by IHC were investigated, as Wide Area Transepithelial Sampling with Three-Dimensional Computer-Assisted Analysis (WATS^3D^), TissueCypher, mutational load analysis (BarreGen), fluorescence in situ hybridization, and DNA content abnormalities as detected via DNA flow cytometry. These tests provide information that cannot be assessed based on morphology alone and may offer more precise surveillance in those cases with an unsatisfactory (IND) diagnosis or LGD at histology [51].

At present, however, in routine practice, morphologic assessment of dysplasia remains the gold standard for evaluating dysplasia.

### 4.5. Molecular Pathway: From Metaplasia to Adenocarcinoma

Despite the peculiarity of its history, the difficulties in determining the cell of origin of BE, and the numerous debates about clinical diagnosis, histological diagnosis, clinical surveillance, etc., in recent years, there is a growing body of evidence that EAC, which is thought to arise in the BE context, shares molecular similarities with GEJ and gastric cancers, with a progressive increase in chromosomal instability phenotype [54]. For this reason, they should be considered as a single entity for clinical trials of neoadjuvant, adjuvant, or systemic therapies. 

Many different oncogenes (c-Myc, Cyclins), growth factors (FGFR, erb-B2/Her2-neu), and tumor suppressors (p53, p16, p15, p27) have been identified during the progression from metaplasia to dysplasia and then EAC [55]. The process of developing esophageal adenocarcinoma involves distinct stages, from BE to primary tumors and advanced metastatic disease. The mucosa in the distal esophagus can be injured by reflux-induced cytokine damage [56,57,58,59]. Bile acid exposure in an acidic environment increases reactive oxygen species in keratinocytes and the metaplastic epithelium in BE [60]. This, in turn, leads to HIF-2α stabilization [61], nuclear translocation, binding to HIF-responsive elements, and the synthesis and release of proinflammatory cytokines. HIF-2α is responsible for regulating the inflammatory response to reflux injury, which is linked to NF-κB signaling via p65 phosphorylation. The activation of NF-κB in the distal esophagus leads to persistent inflammation and triggers the development of intestinal metaplasia through the activation of CDX2. CDX2 plays a crucial role in intestinal differentiation and may be a downstream target of NF-κB, as it contains a binding site for NF-κB [62,63]. Exposure to bile acids in an acidic environment can cause oxidative stress in squamous and metaplastic epithelia in individuals with Barrett’s esophagus (BE). This can lead to DNA damage, particularly double-strand breaks in DNA [62]. Neoplastic progression in the metaplastic segment is mainly driven by TP53 mutations, which can cause a significant increase in genetic abnormalities. TP53 mutations make it easier for other genetic mechanisms to drive carcinogenesis in BE. Loss-of-function mutations in TP53 can lead to exponential growth in several mutations due to impaired mechanisms of DNA repair and apoptosis. Mutations in TP53 serve as a point from which different genetic mechanisms can be realized. In their simplest form, mutational signatures are single-base substitutions in a trinucleotide context. This is reflected in unique patterns of patterns of nucleotide substitutions or larger chromosomal rearrangements. The T>G and T>C substitutions dominate the mutational landscape and is linked with TP53 mutations, increased proliferation, genomic instability, and disease progression [64]. DNA damage, resulting from both internal and external mutational processes during an individual’s life, is recorded in the genome of cancer cells. Obesity has been identified as a major risk factor for esophageal adenocarcinoma due to anatomical factors such as increased abdominal and intraperitoneal adiposity and hiatal hernia formation, which predispose patients to increased gastro-esophageal reflux. Moreover, obesity promotes cancer progression through insulin resistance, inflammation, oxidative stress, and the production of adipokines—hormones associated with fat. Additionally, the signaling of Human Epidermal growth factor receptor 2 (HER2) plays a role in cancer development. The incidence of esophageal cancer can be significantly increased by hyperinsulinemia in the presence of duodenal reflux. In such cases, insulin receptor (IR) and IGF1 receptor (IGF1R) tend to be overexpressed. It appears that IGF1R is responsible for cancer onset through the activation of the ERK1/2 mitogenic pattern. The upregulation of IGF1R and HER2 in hyperinsulinemia could also increase the likelihood of forming IGF1R/HER2 heterodimers, which support cell growth, proliferation, and progression in esophageal carcinogenesis. 

## 5. Future Directions

It is crucial to find more accurate and specific risk factors that can identify the progression of cancer and development biomarkers to ensure the use of the best-targeted therapy. Pathologists play a pivotal role in this process by evaluating tissues starting from the biopsy. The tissue evaluation should provide highly informative and accurate results, avoid potential errors, and maximize the diagnostic yield. In recent years, the development of molecular targeted therapy has driven the scientific and medical community towards evaluating multiple biomarkers useful in this setting: at present, the use of anti-Her2 targeted therapies and immune checkpoint inhibitors is well established in the clinical setting. Multiple biomarkers, including mismatch repair proteins, Her2 [65], and PD-L1 [66,67], assessed via biopsy samples, should be mandatory before initiating the first-line treatment in all patients with gastric cancer [68].

In the current era of biomarker-driven oncology, involving a specialized gastrointestinal pathologist for biomarker assessment in the context of gastroesophageal malignancies is essential.

## 6. Conclusions

In this review, we have attempted to comprehensively summarize the current understanding of Barrett’s esophagus’s biology, pathophysiology, and evolution (BE). Our focus has been on the pathologists’ crucial role in evaluating patients with this disease, which can be challenging and is limited by factors such as poor interobserver agreement, a lack of valid ancillary techniques, and the absence of a universally accepted classification system for lesions. Despite advances in medicine, the diagnosis of BE in 2023 still heavily relies on pathologists’ expertise.

## Figures and Tables

**Table 1:** Diagnosis of Barrett’s esophagus (BE) according to different guidelines.

Society	Length Criteria	Landmark	Intestinal Metaplasia
ASGE	Any	PMGF	Required
ACG	≥1 cm	PMGF	Required
AGA	Any	PMGF	Required
ESGE	≥1 cm	PMGF	Required
BSG	≥1 cm	PMGF	Not Required
APAGE	≥1 cm	PMGF	Not Required
JES	Any	DEPV	Not Required

ASGE—American Society for Gastrointestinal Endoscopy; ACG—American College of Gastroenterology; AGA—American Gastroenterological Association; ESGE—European Society of Gastrointestinal Endoscopy; BSG—British Society of Gastroenterology; APAGE—Asian Pacific Association of Gastroenterology; JES—Japanese Esophageal Society; PMGF—proximal margin of gastric folds; DEPV—distal end of palisade vessel.

**Table 2:** The Vienna and Reid classifications of dysplasia in Barrett esophagus [43].

Vienna	Reid
Negative for neoplasia/dysplasia	Negative for dysplasia
Indefinite for neoplasia/dysplasia	Indefinite for dysplasia
Non-invasive low-grade neoplasia (low-grade adenoma/dysplasia)	Low-grade dysplasia
Non-invasive high-grade neoplasia	High-grade dysplasia
▪ High-grade dysplasia ▪ Non-invasive carcinoma (carcinoma in situ) ▪ Suspicious for invasive carcinoma	
